# Imaging mitochondria through bone in live mice using two-photon fluorescence microscopy with adaptive optics

**DOI:** 10.3389/fnimg.2023.959601

**Published:** 2023-02-16

**Authors:** Tianyi Zheng, Adrian R. Liversage, Kayvan F. Tehrani, Jarrod A. Call, Peter A. Kner, Luke J. Mortensen

**Affiliations:** ^1^School of Electrical and Computer Engineering, University of Georgia, Athens, GA, United States; ^2^School of Chemical, Materials and Biomedical Engineering, University of Georgia, Athens, GA, United States; ^3^Biophotonics Imaging Laboratory, The University of Illinois Urbana-Champaign, Urbana, IL, United States; ^4^Department of Physiology and Pharmacology, University of Georgia, Athens, GA, United States; ^5^Regenerative Bioscience Center, Rhodes Center for ADS, University of Georgia, Athens, GA, United States

**Keywords:** two-photon fluorescence microscopy, aberration, adaptive optics, mitochondria, mouse cranial bone

## Abstract

**Introduction:**

Mitochondria are extremely important organelles in the regulation of bone marrow and brain activity. However, live imaging of these subcellular features with high resolution in scattering tissues like brain or bone has proven challenging.

**Methods:**

In this study, we developed a two-photon fluorescence microscope with adaptive optics (TPFM-AO) for high-resolution imaging, which uses a home-built Shack-Hartmann wavefront sensor (SHWFS) to correct system aberrations and a sensorless approach for correcting low order tissue aberrations.

**Results:**

Using AO increases the fluorescence intensity of the point spread function (PSF) and achieves fast imaging of subcellular organelles with 400 nm resolution through 85 μm of highly scattering tissue. We achieved ~1.55×, ~3.58×, and ~1.77× intensity increases using AO, and a reduction of the PSF width by ~0.83×, ~0.74×, and ~0.9× at the depths of 0, 50 μm and 85 μm in living mouse bone marrow respectively, allowing us to characterize mitochondrial health and the survival of functioning cells with a field of view of 67.5× 67.5 μm. We also investigate the role of initial signal and background levels in sample correction quality by varying the laser power and camera exposure time and develop an intensity-based criteria for sample correction.

**Discussion:**

This study demonstrates a promising tool for imaging of mitochondria and other organelles in optically distorting biological environments, which could facilitate the study of a variety of diseases connected to mitochondrial morphology and activity in a range of biological tissues.

## 1. Introduction

Mitochondria are intracellular organelles with 0.5 to 10 μ*m* diameter that drive energy production processes through the respiratory chain by oxidative phosphorylation (Siesjo, 1978; Siegel et al., 1981). They play a fundamental role in numerous physiological processes of critical importance in tissue homeostasis and repair, such as cell differentiation (Folmes et al., 2012), apoptosis (Miao et al., 2014), signal transduction (Xu et al., 2016), reactive oxygen species generation (Murphy, 2009), and maintenance of healthy organ function (Gropman, 2004). Their function is highly dynamic and reflected in mitochondrial network structure, and imaging technologies are therefore essential to understand physiological mitochondrial processes in health and disease. High energy requirement tissues such as the brain and bone marrow are especially dependent on carefully orchestrated mitochondrial maintenance and activity.

Over the last decades, the evaluation of live tissue dynamics at cellular resolution using intravital imaging has transformed the biological understanding of organ function at a single cell level. In the bone, this has significantly advanced the scientific understanding of vascular dynamics, stem cell biology, and bone homeostasis and regeneration (Lo Celso et al., 2009; Spencer et al., 2014; Itkin et al., 2016; Wilk et al., 2017; Christodoulou et al., 2020). In the brain, intravital imaging has generated unique insight into brain circuitry and processing, brain cancer, brain trauma, and degenerative diseases (Andermann and Kerlin, 2010; Shih et al., 2012; Ricard, 2014; Yang et al., 2018; Calvo-Rodriguez et al., 2019; Chen et al., 2019; Hu et al., 2021). However, one of the most serious obstacles to imaging is the poor penetration depth of intravital optical microscopy. Single photon imaging with confocal detection is a common approach that uses visible light for fluorescence excitation (400–650 nm), where light penetration is attenuated by absorption and scattering of skull bone and tissues (Shi et al., 2016; Wang et al., 2018). To extend imaging depth, high energy pulses of near-IR excitation light (760–1,080 nm) can be tightly focused to create a non-linear two-photon absorption process using standard fluorophores. Two-photon imaging extends the attainable imaging depth to 500 μm−1 mm of brain tissue and up to ~150 μm in highly scattering bone (Denk and Strickler, 1990; Hell, 1992; Xu, 1996; Callis, 1997; Diaspro et al., 2006; Sinefeld et al., 2015). However, non-homogeneous wave propagation through these irregular and highly distorting turbid media induces high magnitude phase deviations in the wavefront (Tehrani et al., 2017a) that dramatically reduce image resolution even at moderate depths. Therefore, cranial windows and skull-thinning methods are commonly adopted to improve optical access (Jeong and Tsai, 2013; Yang et al., 2013; Chen et al., 2021). However, the surgery increases the risk of tissue inflammation and may cause stress that could alter biological function in the target tissues (Li and Baran, 2014).

One way to overcome this challenge is with adaptive optics (AO). An AO system typically consists of a deformable mirror conjugated to the back pupil plane of a microscope, and either wavefront sensor or image-based sensorless wavefront estimation methods to correct aberrations and improve the resolution, which can improve *in vivo* imaging in animal models (Albert et al., 2000; Wright et al., 2005; Rueckel and Mack-Bucher, 2006; Débarre et al., 2009; Tao et al., 2013a; Kong, 2015a,b; Wang et al., 2015; Kong and Tang, 2016). Wavefront sensor approaches include Shack–Hartmann wavefront sensors with auto-fluorescent or near-IR guide stars (Tao et al., 2013a; Wang et al., 2015), coherence gated wavefront sensing (Rueckel and Mack-Bucher, 2006), and image-based methods that use information from acquired images to remove wavefront distortions (Marsh and Burns, 2003; Débarre et al., 2009; Kong, 2015a,b; Kong and Tang, 2016). Wavefront sensorless approaches usually estimate an initial error and through an iterative scheme converge to an optimized solution based on intensity metrics (Albert et al., 2000; Wright et al., 2005; Booth, 2006). Recently, non-linear guide stars with Shack–Hartmann measurements of wavefront aberrations have yielded an accurate measurement of low-order tissue aberrations that proves to be useful to extend imaging depth in biological samples but require long integration times and has a small effective field of view (Aviles-Espinosa et al., 2011; Tao et al., 2013b). In an alternative wavefront sensorless approach using rapid anisotropic aberration correction, the adaptive correction element was conjugated to the turbid layer instead of the focus, with the goal of increasing the size of the isoplanatic patch (Park and Sun, 2015). Our bone marrow imaging occurs within an extended scattering layer, so such an approach is not appropriate.

In this study, we calculate and correct both the system aberration and the sample aberrations caused by mouse cranial bone and brain tissue to improve imaging for dynamic mitochondria localization. We demonstrate that low-order aberration correction provides a significant improvement when imaging through the bone into the bone marrow. We first compensate for the aberrations of our microscope system using a sensor-based AO algorithm, and then we compensate for the aberrations of mouse cranial bone by using a Zernike-mode-based sensorless AO algorithm because it requires comparatively less signal to optimize and create an improved wavefront profile. We use our two-photon fluorescence microscope with adaptive optics to image mitochondria in mouse cranial bone marrow and brain. We also find and evaluate the threshold and performance for intensity-based sample correction. This work shows that after AO correction, the fluorescence intensity of the point spread function (PSF) is improved, and the resolution of images is significantly improved when imaging through intact mouse cranial bone into the bone marrow, allowing us to characterize mitochondrial health and dynamics of functioning cells deep in tissue.

## 2. Methods

### 2.1. Preparation of fluorescent beads stack in gel

A total of 200nm yellow-green, fluorescent beads (ThermoFisher Scientific F8811) were diluted to a ratio of 1:200 in 2.0% agarose. Then, 0.2 grams of agarose powder (Bio-Rad, Certified Molecular Biology Agarose 1613101) were added to 10 milliliters of DI (Deionization) water to make the mass concentration 2.0%. Next, the agarose solution was heated in a microwave for intervals of 45 s. This process was continued until the agarose became a gel mixture. After 1–2 min passed for the agarose gel to cool down to a safe handling temperature, the agarose gel was ready to hold the beads in place for imaging analysis. In total, 2.0 ml of beads were added to 400 ml of agarose to achieve the 1:200 ratio. After drying completely, the Petri dish was then placed on the stage of the TPFM-AO system and DI water was added for imaging with the water dipping objective.

### 2.2. Mouse imaging

For mouse intravital imaging, we used a transgenic mouse model ubiquitously expressing mitochondrial-targeted Dendra2 green monomeric fluorescent protein (Jackson Laboratory, #018385) as previously described (Pham et al., 2012; Southern et al., 2019). The mouse was initially anesthetized using 4% isoflurane (100 ml/min oxygen flow) and restrained using a 3D printed stereotaxic holder. The holder is similar to those used in previously published works by several groups when studying cell dynamics in the brain and skull (Fried et al., 2001; Lo Celso et al., 2009; Leuschner et al., 2015; Turcotte et al., 2017; Tehrani et al., 2019b) and serves to secure and stabilize the mouse skull while reducing mechanical coupling with the trunk of the body so that breathing movement artifacts are reduced. Five minutes before making an incision, 50 μl of 0.25% bupivacaine was locally applied as analgesia. An incision was made on the scalp from between the eyes toward both ears to make a flap. The periosteum layer was removed, and the area of imaging was cleaned using a cotton swab; immediately sterile phosphate buffered saline (PBS) was applied to the incision site. The animal was placed under the microscope objective and sterile PBS was added to fill the gap between the skull and the objective lens. The rate of isoflurane was then reduced to 1.4% during imaging. For vasculature imaging, a 20 μl dose of 70 kDa rhodamine-B dextran (Nanocs) was administered through retro-orbital injection before making an incision. After the intravital imaging session, mice were euthanized using CO_2_ and cervical dislocation. Immediately after sacrifice, the brain was extracted and mounted in a Petri dish using 2.0% agarose for mitochondrial imaging. All animal procedures and experiments were approved by the UGA Institutional Animal Care and Use Committee (IACUC).

### 2.3. Optical setup

**Figure 1:**
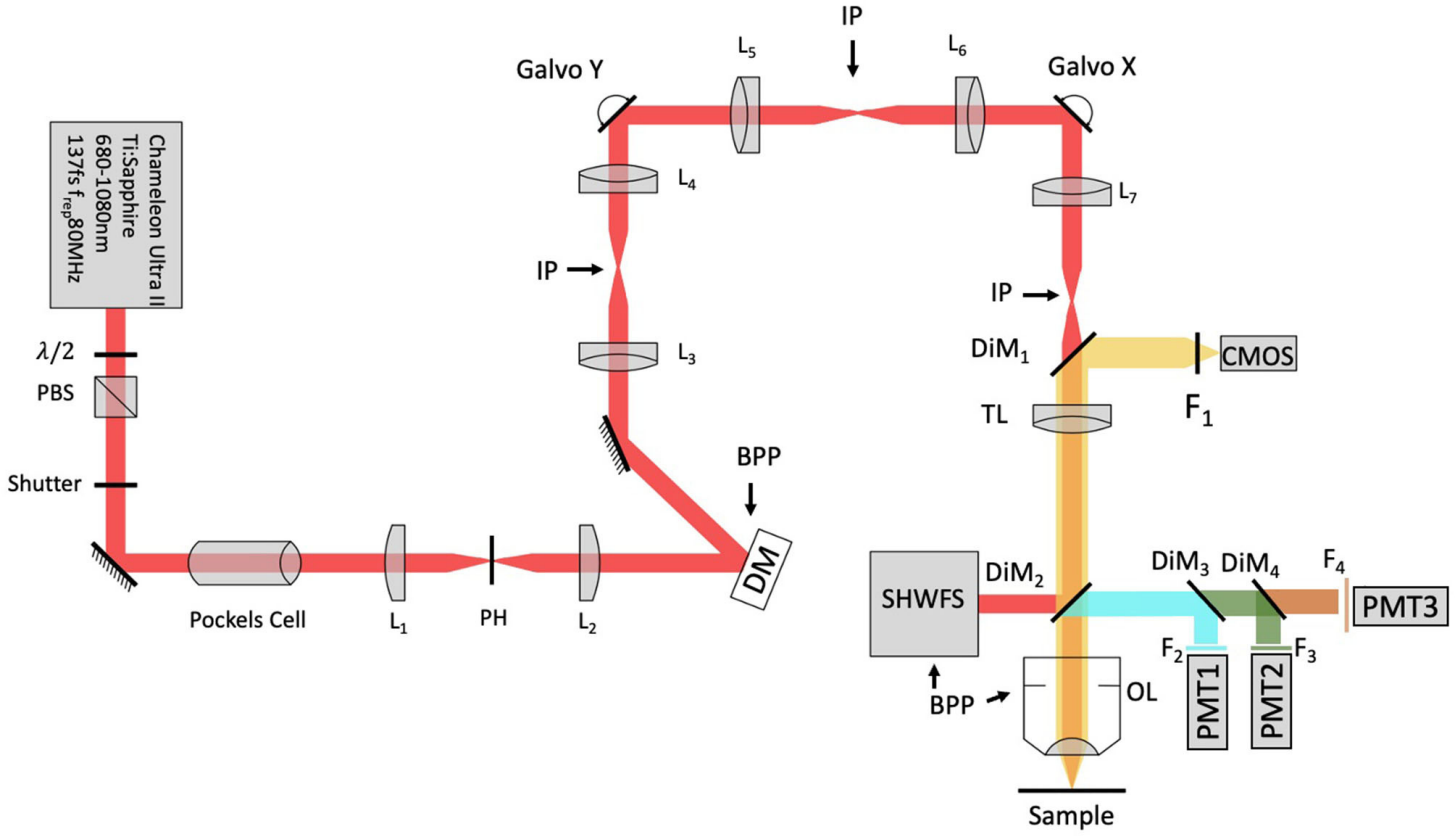
Modified system design with SHWFS. BPP, Back pupil plane; CMOS, scientific camera; DG, diffraction grating; DiM, Dichroic mirror; DM, deformable mirror; F, Filter; IP, image plane; L, Lens; OL, Objective lens; PBS, Polarizing beam splitter; PH, pinhole; PMT, photon multiplier tube; SHWFS, Shack, Hartmann wavefront sensor; TL, tube lens.

**Figure 2:**
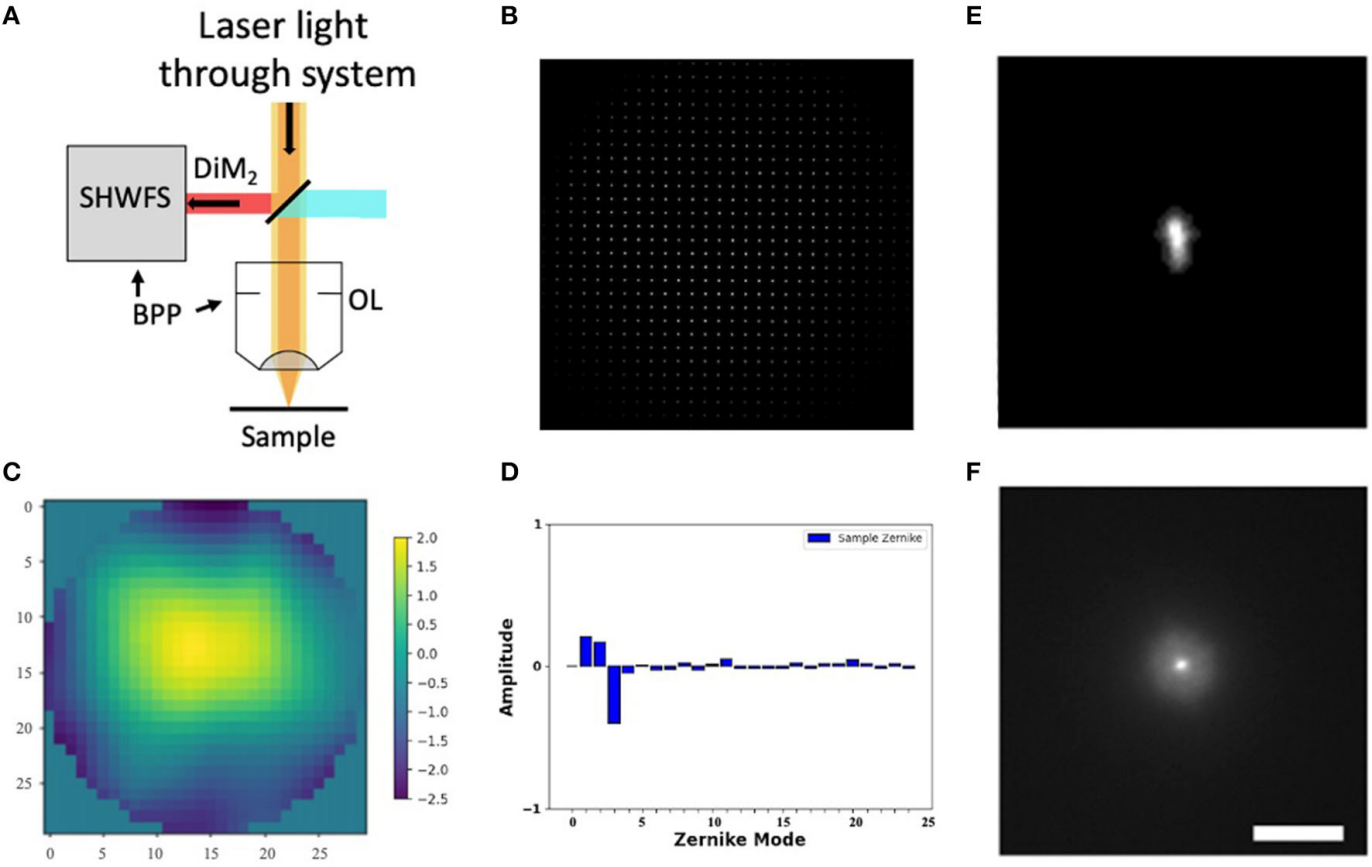
Shack-Hartmann wavefront sensor. **(A)** SHWFS used to measure the total system aberrations **(B)** Image of the Shack-Hartmann spots on the SHWFS camera. **(C)** Corrective wavefront (μm). **(D)** Singular value decomposition of the Zernike modes. **(E, F)** PSF before and after system correction. Scale bar = 10 μm.

A schematic of the adaptive optics two-photon fluorescence microscopy (AO-TPFM) system is shown in Figure 1, based on our previously published work (Tehrani et al., 2017b, 2021). The optical setup consists of a Chameleon Ti:Sapphire laser producing 680 to 1,080 nm 137 fs pulses of energy, with power at the source modulated using a half-waveplate and a polarizing beam splitter. The laser beam is recollimated and passed through a Pockels cell (Conoptics) for rapid intensity modulation during imaging. The beam is then expanded using a telescope with a pinhole in the focus to create a more uniformly Gaussian beam profile. The Pockels cell is used to block the beam during flyback, as well as correct beam intensity for the resonant scanner. The deformable mirror (DM, Alpao DM97-15) placed conjugate to the back pupil plane has a continuous face sheet and 97 actuators and is used for low-order AO wavefront correction. The DM is inserted into the beam path with two optical relay systems. Beam scanning is done by a Sutter instrument MDR-R box that houses a fast resonant galvo and a slow galvo scanner for horizontal and vertical sweeping, respectively. Both scanners are from Cambridge technology and are placed very close to each other to reduce astigmatism, with heatsinks. The scanned beam is relayed using two achromatic doublets—serving as the scanning and tube lenses, respectively—to the 60 × 1.00 NA water immersion objective lens (Nikon, MRD07620). The back-propagated emission light from the sample is separated from the excitation light using a dichroic mirror DiM_1_ (Semrock FF705-Di01) and sent to an sCMOS camera (ANDOR, Zyla Scientific CMOS) which is used to look at the PSF shape or separated with a dichroic mirror DiM2 (Semrock FF705-Di01) and sent to the photon multiplier tubes (PMT) from Hamamatsu (H10770-40). DiM3-4 (Semrock FF552-Di02, and FF409-Di03-25^*^36) and filters F2-4 (Semrock 571/72 nm, 509/22 nm, and 390/18 nm) were used to separate each spectral channel to capture signals from two photon fluorescence (TPF), green fluorescent protein (GFP), and second harmonic generation (SHG) of collagen, respectively. A home-built Shack-Hartmann wavefront sensor (SHWFS) was used, with a LabVIEW-based control and measurement software to measure the total system aberrations (red arrow shows beam direction) just before the objective lens (Figure 2A). Another LabVIEW-based control and measurement software was used for full AO correction. The MATLAB^®^-based open-source software, Scanimage (Pologruto and Sabatini, 2003) was employed to control the microscope after the correction.

### 2.4. Sensor-based system aberrations

Before measuring and correcting the aberrations of the biological samples, we first compensated for system aberrations using a sensor-based AO algorithm. The lenslet array creates spots in the image whose displacement versus an internal reference guidestar allows calculation of wavefront distortions (Figure 2B).

We use Eq. (1) to decompose the calculated wavefront into the Zernike modes (Zhao, 2007):


(1) $$c_{i}=\frac{1}{\pi }\int_{0}^{1}\int_{0}^{2\pi }\psi_{i}\left(\rho ,\theta \right)Z_{i}\left(\rho ,\theta \right)\rho d\theta d\rho ,$$

where Z is the Zernike mode of order *i* and **c**_**i**_ is the coefficient of the mode **Z**_**i**_. Equation (1) yields a complete Zernike coefficient set that could be applied to a DM for correction. We take modes 5 to 37 [using Noll's ordering (Noll, 1976) of the Zernike modes, up to order 4] into consideration because these modes can be corrected by the DM. To find the corrected wavefront shape, we do a summation such that constructed phase equals


(2) $$\psi_{c}\left(\rho ,\theta \right)=\exp\left(-j\frac{2\pi }{\lambda }\sum_{\text{i}}c_{i}Z_{i}\left(\rho ,\theta \right)\right)$$

The root-mean-square (RMS) wavefront error that is corrected by the Zernike modes 4 to 37 (piston, tip, and tilt are not included) is calculated by $\sigma =\;{\left[\underset{i}{\sum }c_{i}^{2}\right]}^{1/2}$.

We perform one DM correction at each focal plane by scanning for a fluorescent signal and then optimizing the wavefront (Tehrani et al., 2019a). The wavefront of our excitation beam shows <2 waves of distortion, with Zernike decomposition of the wavefront identifying the strongest contributions from tip (Z2), tilt (Z3), and defocus (Z4) (Figures 2C, D). After applying the Shack-Hartmann wavefront correction, the PSF in the sample plane has a near diffraction-limited Gaussian shape (FWHM = 350 nm).

### 2.5. Sensorless sample aberration correction based on sum or max intensity

Wavefront aberrations are the difference in phase or optical path length from the ideal (e.g., spherical, or planar) form Neil et al. (2000), which can be caused by light propagation through an inhomogeneous medium like biological tissue. According to the Zernike mode equation, different combinations of the Zernike coefficients in a phase distribution at the back-pupil plane can alter the point spread function at the focal plane. If used to reconstruct the wavefront phase distribution with proper Zernike modes and coefficients by a DM placed conjugate to the back-pupil plane, this principle can compensate for the aberrations induced by tissue.

Sensorless AO uses the signal obtained with the microscope as an input for an algorithm that estimates the optical aberrations present in the system. Our implementation of sensorless AO is depicted in the flowchart in Supplementary Figure S1. A series of PSFs is acquired with different Zernike aberration modes applied to a DM conjugate with the back pupil plane. To determine the optimal value for each Zernike mode, different values of aberration are applied to the DM. We evaluated 10 or 15 orders of Zernike modes (tip, tilt, and defocus excluded), which in our preliminary experiments using 10 or 15 modes for correction provided 90% of the enhancement found when including higher order modes. An image quality metric (e.g., max intensity) is then selected and evaluated for each image. Then a parabolic function is fitted to the measured points and the mode coefficient corresponding to the estimated peak is applied as the correction. Subsequent modes are corrected in a similar manner to achieve convergence of the wavefront.

### 2.6. Sample correction parameter selection

**Figure 3:**
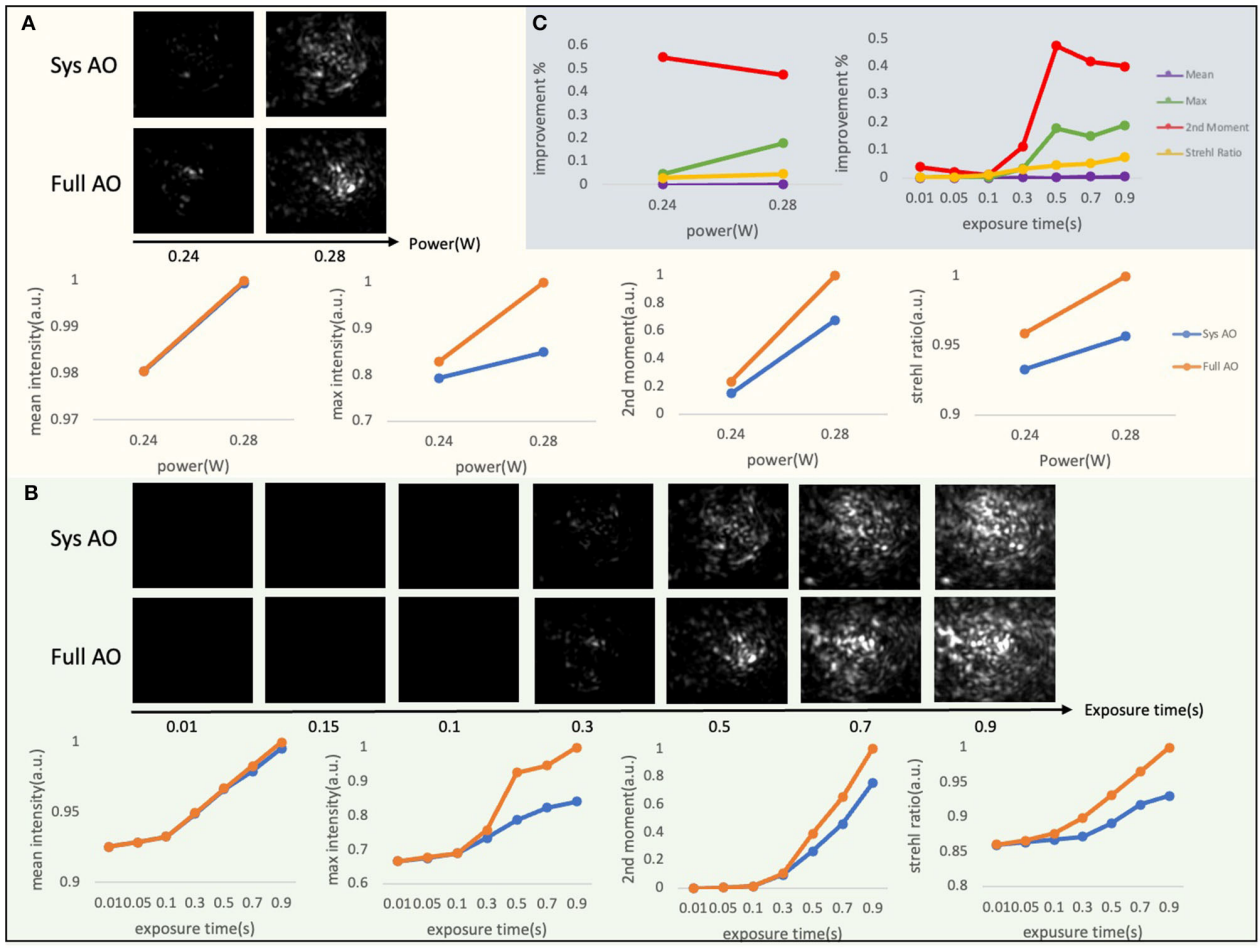
Intensity-based sample correction. Both laser power before the objective lens **(A)** and camera exposure time **(B)** could affect the results of sample correction. Section **(C)** shows the improvement percentage calculated by (full AO—system AO)/system AO intensity. The percent changes between the uncorrected and corrected value reach a constant.

During preliminary aberration correction experiments, we observed that the degree of enhancement achieved in sensorless sample correction has a dependence on the initial intensity conditions in low signal environments. Since a low starting signal is common when attempting to use AO, we explored the sensitivity of our correction strategy to different background noise and signal levels by modulating the laser power before the objective lens (Figure 3A) and the camera exposure time (Figure 3B). These measures both strongly influence the signal to background ratio of the PSF images used in the sample correction for a GFP mouse skull. We used two power levels at the sample, a commonly used average power on the sample for imaging 50–100 μm deep in tissue, and the maximum average power that we experimentally observed not to cause visible damage to the sample within our image acquisition time; and a range of integration times for each Zernike mode measure from 10 ms to 1 s. At each condition, we evaluated several measures of PSF quality, including the mean intensity, max intensity, second moment, and Strehl ratio. To calculate the second moment, the k-th central moment of a data sample is defined as: $m_{k}=\frac{1}{n}\overset{n}{\underset{i=1}{\sum }}{\left(x_{i}-\bar{x}\right)}^{k}$, where *n* is the number of samples and $\bar{x}$ is the mean. The Strehl ratio is defined as $S=e^{-\frac{4\pi^{2}\sigma^{2}}{\lambda^{2}}}$, where σ is the root mean square deviation of the wavefront, λ is the wavelength. In our situation, the experimental Strehl ratio is defined as the ratio of the peak aberrated image intensity from a point source compared to the maximum attainable intensity using an ideal optical system limited only by diffraction over the system's aperture.

According to Figure 3B, although we are not getting improvement based on mean intensity, we achieve improvement based on max value, second moment, and Strehl ratio of PSF when the exposure time is larger than 0.5 s, with the percent changes between the uncorrected and corrected value reaching a constant soon after (Figure 3C). Therefore, we find that by performing our TPFM-AO approach at two distinct signal levels using mean or max intensity of PSF when the improvement percentage of two of the measurements are almost the same, the sample correction has achieved its maximum improvement performance. In future work, we will further explore alternate correction metrics (e.g., second moment) for *in vivo* situations.

## 3. Results and discussion

### 3.1. *In vitro* tissue mimic with AO correction for submicron bead imaging

**Figure 4:**
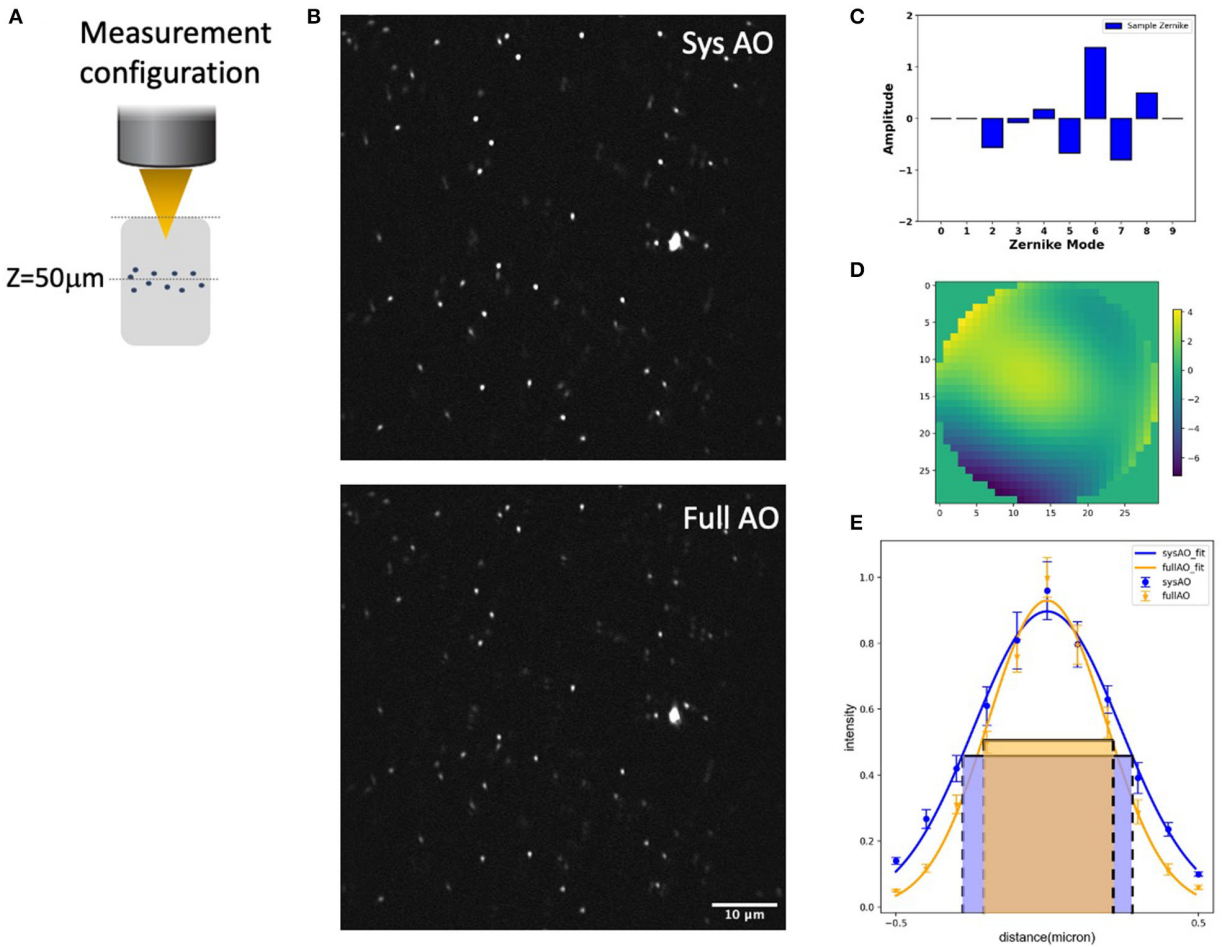
System and mean-intensity-based full AO on 0.2 μm beads **(B)** at 50 μm depth in an agarose gel **(A)**. Zernike mode decomposition of the wavefront **(C)**. Wavefront after full AO (μm) **(D)**. Spot size **(E)**. The red and green boxes represent the FWHM of each intensity profile. The FOV is 67.5 × 67.5 μm. Data are represented as mean +/– standard deviation for each measurement. Scale bar = 10 μm.

We evaluated the experimental resolution improvement in a tissue phantom of the TPFM-AO microscope by measuring the full-width at half-maximum (FWHM) of the intensity profile of 0.2 μm beads embedded in a gel using a 960-nm laser excitation wavelength (Figure 4). Beads were embedded in a 5 mm thick 2% agarose gel and imaged at 50 μm depth (Figure 4A) to mimic a distorting tissue environment. We imaged the sample with system correction on, and then used a sensorless approach to compensate for the aberrations induced by the gel (Figure 4B). The full AO correction approach shows relatively high values of astigmatism (Z6) and coma (Z7) and yields the wavefront shown in Figures 4C, D. AO improved the full width at half maximum of the detected bead fluorescence with the average FWHM Gaussian fit of 10 measured 0.2 μm radius 2-photon excited fluorescent beads improving from 0.538 ± 0.03 μm to 0.408 ± 0.03 μm after sample correction (Figure 4E).

### 3.2. AO correction enables high-resolution imaging of mitochondria organelle morphology in the mouse brain

**Figure 5:**
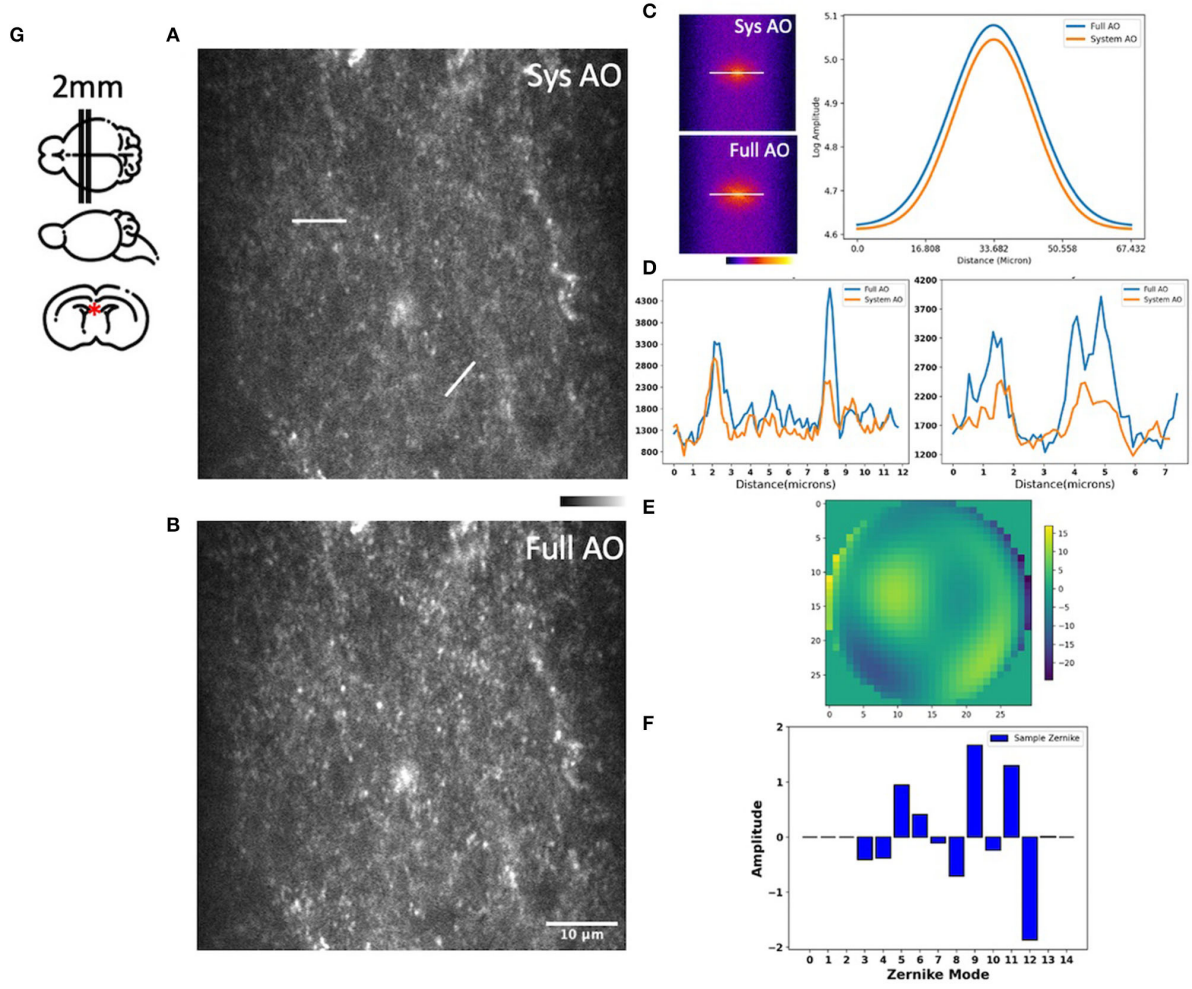
Images of the hippocampus of a young mouse brain with system AO **(A)** and with mean-intensity-based full AO **(B)**. Corresponding FFTs of the brain image in log scale and line profiles **(C)**. Corresponding signal profiles along the white lines, the y-axis is intensity **(D)**. Wavefront after full AO (μm) **(E)**. Singular value decomposition of the Zernike modes **(F)** for sample correction. The hippocampus of young mouse was imaged at the red asterisk **(G)**. The FOV is 59.32 × 59.32 μm. Scale bar = 10 μm.

We then measured and corrected aberrations for Dendra-2 mouse brain mitochondria using a 780 nm laser excitation wavelength. Immediately after sacrifice, the mouse brain was cut into 2 mm thick slices, then embedded in 3% agarose to prevent movement. For imaging, the brain was immersed in phosphate buffered saline (PBS). Two photon fluorescence images were acquired in the hippocampal region, as indicated by a red asterisk in our brain diagram (Figure 5G). We found tissue wavefront distortions (Figure 5E) and aberrations due to the shape and high refractive index of the brain; mostly astigmatism (Z5, Z11), trefoil (Z9), and spherical (Z11) (Figure 5F). After AO, the images had improvements in mitochondrial intensity and sharpness laterally and, especially, axially by correcting the aberrations (Figures 5A, B). In the spatial frequency space (Figure 5C), the resolution improvement gained through aberration correction led to a substantial increase in the magnitude of high spatial frequency components, which indicates a sharper image with more fine detail. Signal profiles in the axial plane along the white lines show improved intensity (Figure 5D).

### 3.3. AO correction enables mitochondria imaging deep in mouse bone marrow *in vivo*

**Figure 6:**
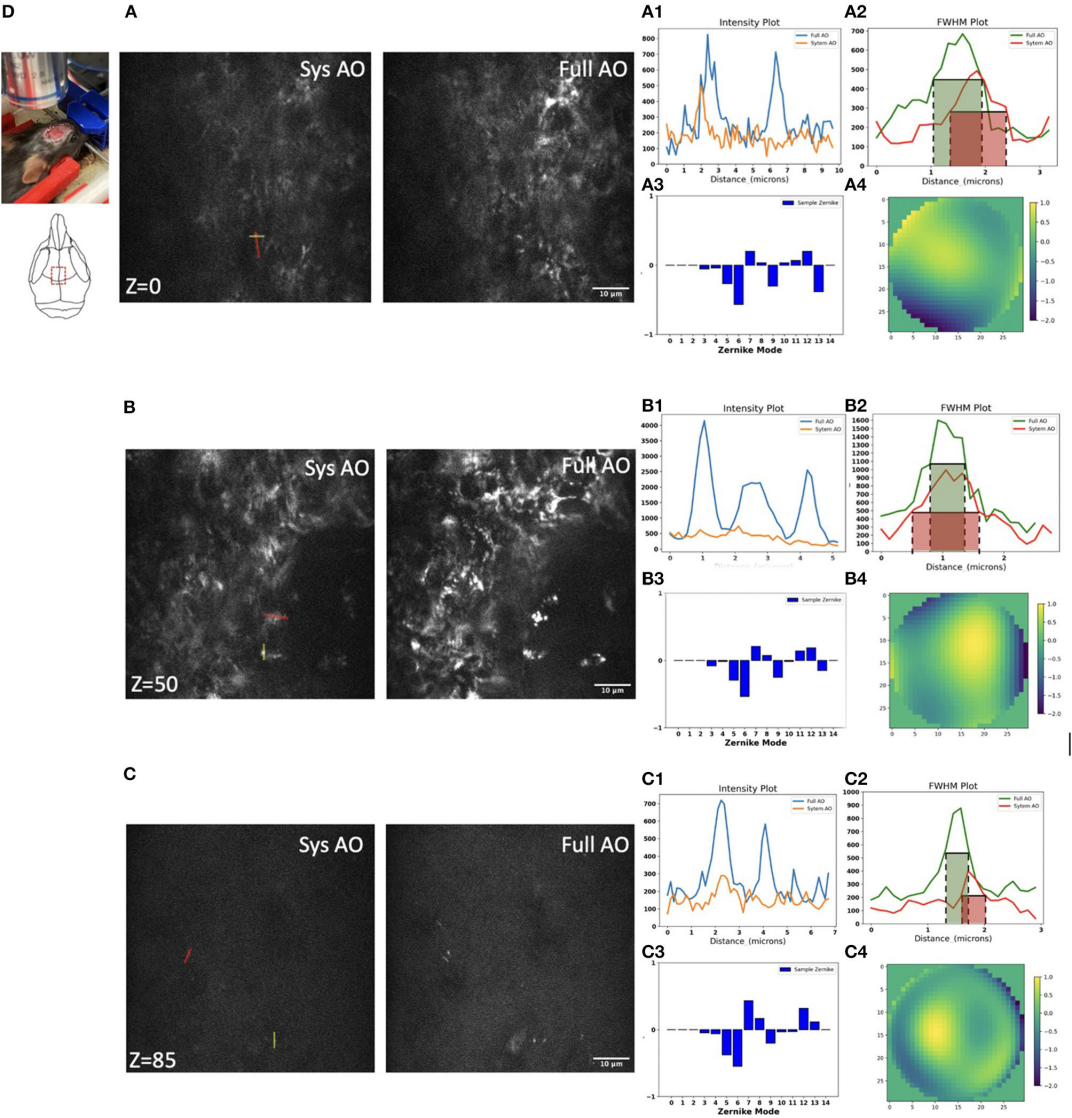
Dynamic imaging of GFP-mitochondria mouse bone marrow at the depth of 0, 50, and 85 μm with system AO (left) and with mean-intensity-based full AO (right) **(A–C)**. Wavefront after sample correction (μm) **(A4–C4)**. Corresponding signal profiles of the red line **(A1–C1)** and FWHM profiles of the yellow line **(A2–C2)** and corresponding Zernike modes **(A3–C3)** for full AO. The y-axis for signal profiles and FWHM profiles is intensity. Images were acquired at the red square at 0, 50, and 85 μm depth **(D)**. The red and green boxes represent the FWHM of each intensity profile. The FOV is 67.5 × 67.5 μm. Scale bar = 10 μm.

We next evaluated mitochondrial imaging through the outside layer of cranial bone into the bone marrow using the Dendra-2 mouse with 780 nm laser excitation wavelength. We evaluated 15 orders of Zernike modes to correct aberrations of the bone marrow *in vivo* because in preliminary experiments this achieved the best improvement of the PSF intensity and shape. We found improvements in image intensity and resolution for *in vivo* TPF imaging of mitochondria in bone marrow at the depth of 0, 50, and 85 μm (Figures 6A–C). Signal profiles along the red line show ~1.55×, ~3.58×, and ~1.77 × intensity increases using sample AO correction at the depths of 0, 50, and 85 μm separately (Figures 6A1–C1). The FWHM with full AO was enhanced by ~0.83×, ~0.74 × and ~0.9 × at the depth of 0, 50, and 85 μm separately (Figures 6A2–C2). At the surface of the bone, we observed aberrations due to the shape and high refractive index of the bone; mostly astigmatism (Z6), trefoil (Z9), and secondary astigmatism (Z13) (Figure 6A3). When we go 50 μm deep in the bone marrow, the aberrations of primary astigmatism and trefoil are lower magnitude, but secondary astigmatism (Z13) remains (Figure 6B3). When we reach the opposite side of the bone marrow at a depth of 85 μm, aberrations are mainly coma (Z7) and secondary astigmatism (Z13) resulting from the curved interior surface of the bone (Figure 6C3). The corrective wavefront after full AO is shown in Figures 6A4–C4.

**Figure 7:**
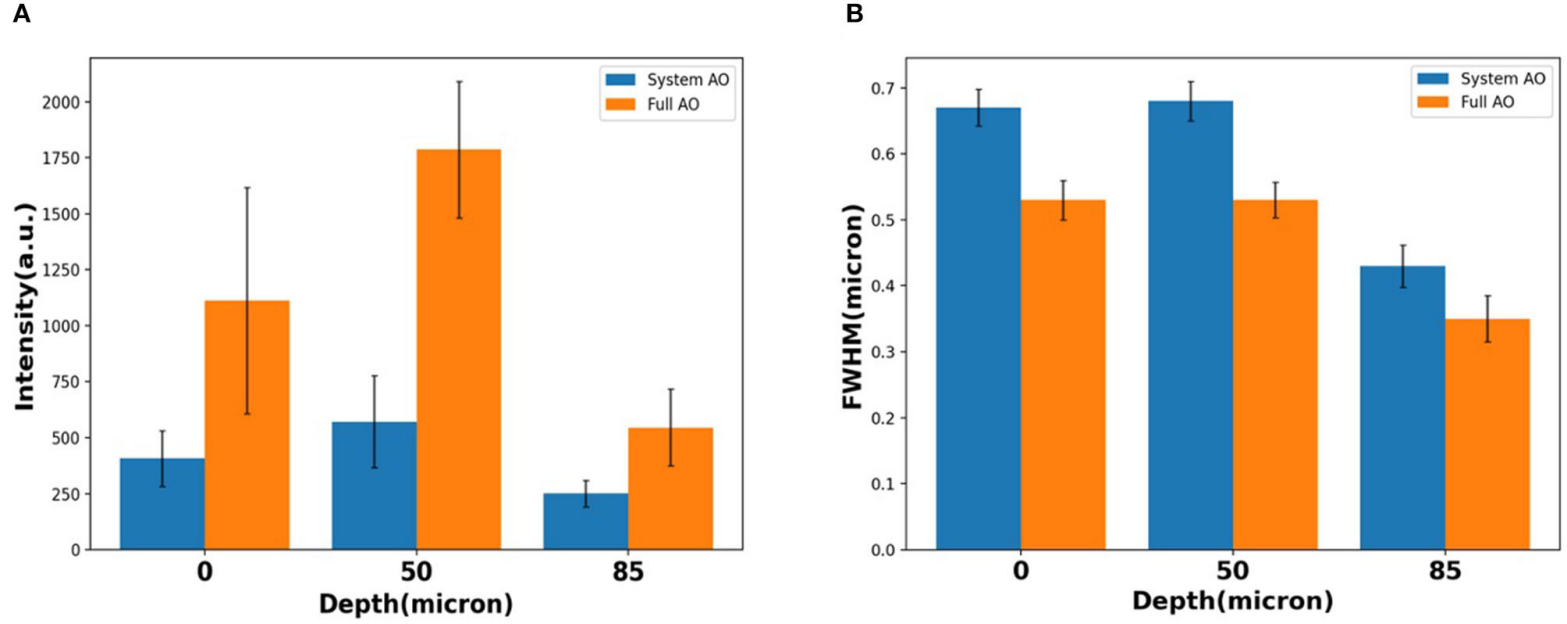
Loss of resolution and maintenance at the depth of 0, 50, 85 μm with system AO and with full AO in intensity **(A)** and FWHM **(B)**. The error bar shows the standard deviation of each measurement.

When averaging across a whole image, the signal intensity showed ~2.73×, ~3.13×, and ~2.18 × intensity increases over TPFM with system AO correction at 0, 50, and 85 μm depth (Figure 7A). The average detected FWHM improvement was ~0.79 × (1.0 μm before and 0.79 μm after), ~0.78 × (0.8 μm before and 0.62 μm after), and ~0.81 × (0.45 μm before and 0.36 μm after) at the depth of 0, 50, and 85 μm respectively (Figure 7B). In order to evaluate the effect of AO on other emission wavelengths and confirm that the 85 μm depth was through the bone marrow and into the bone on the other side, we captured second harmonic generation images with a bandpass filter of 390/18 (Supplementary Figure S2), finding that the FFT spectrum contains more high frequency information after full AO.

We also evaluated another multichannel dynamic *in vivo* bone marrow sample using the Dendra2 mitochondria mouse and co-labeling the blood vasculature using a rhodamine-B dextran conjugate. This stain required correction of the excitation beam using 840 nm excitation wavelength and emission with a 585/40 nm filter. We achieved improved imaging with full AO correction at three different depths of 30, 50, and 70 μm, demonstrating the utility of the system for AO in dynamic samples at multiple excitation and emission wavelengths (Supplementary Figure S3).

### 3.4. AO correction for dynamic mitochondria evaluation in mouse bone marrow

**Figure 8:**
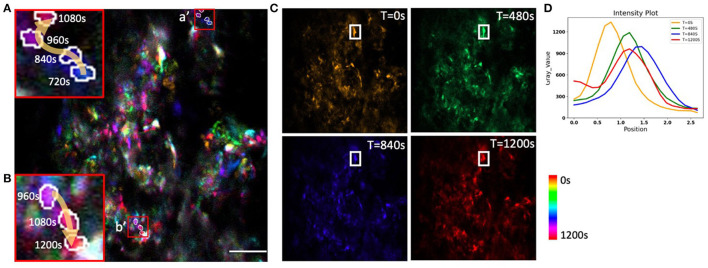
Time-coded pseudo color max projection of time lapse imaging of GFP-mitochondria mouse bone marrow at the depth of 40 μm with mean-intensity-based full AO. **(A/a', B/b')** shows an overview of mitochondria traveling in the bone marrow. **(C)** shows different time points. **(D)** shows mitochondria fluctuations. The y-axis is intensity. The FOV is 67.5 × 67.5 μm. Scale bar = 10 μm.

To evaluate the potential of AO correction to longitudinally monitor mitochondrial organelle dynamics in the bone, we corrected tissue aberration at a single plane 40 μm deep in the bone marrow and then performed time lapse imaging for a total of 20 min (Supplementary Video S1). We quantified the temporal change in the cell mitochondria position (Figure 8) and observed differences in mitochondrial movement rates and trajectories (Figures 8A/a′, B/b′). We found minimal intensity reduction over the imaging session, suggesting that there was little photobleaching or change in the tissue aberrations over 20 min (Figures 8C, D). A variety of cell and mitochondria trajectories are clearly present within relatively small regions of the bone marrow, which is not surprising given the high cellular density and diversity of cell types.

## 4. Conclusion

In summary, we calculated and corrected both the system aberrations and the sample aberrations caused by highly scattering brain tissue and bone with both a sensor-based approach using a Shack–Hartmann wavefront sensor and a sensorless AO approach using the PSF intensity as a metric. We demonstrate that low-order aberration correction provides a significant improvement when imaging through the bone into the living bone marrow. We find our TPFM-AO system increases the fluorescence intensity of the PSF and achieves fast imaging of subcellular organelles with ~400 nm resolution. We also achieved close to a 2 fold increase in intensity and a reduction in PSF width using AO in living mouse bone marrow, allowing us to better characterize mitochondrial health and the survival of functioning cells. We also determined the criteria for stopping iterative sensorless AO correction, finding that once the improvement percentage of two measurements is near constant, sample correction has reached its best performance. This helps us more rapidly and efficiently correct the PSF. This AO approach could be used for the study of the dynamics of other organelles and is applicable to a wide range of biological tissues. In the future, we will further explore alternate correction metrics (e.g., second moment) for *in vivo* situations and use our system to study the transient functional responses in a cell population deep in the bone marrow previously unreachable by optical microscopy. This could transform MSC therapeutic approaches and enable new fundamental biological understanding in the musculoskeletal and neural fields.

## Data availability statement

The raw data supporting the conclusions of this article will be made available by the authors, without undue reservation.

## Ethics statement

The animal study was reviewed and approved by E.L. Rhodes Center for Animal and Dairy Science, Athens, GA.

## Author contributions

TZ was involved in conceptualization, design of the optical system, development of the algorithms and the LabView code, data analysis, project management, and writing. ARL was involved in conceptualization, design of the optical system, and project management. KFT was involved with the initial conceptualization, design of the optical system, and development of the algorithms and the LabView code. JAC was involved in investigation and writing. PAK and LJM were involved in conceptualization, investigation, and writing. All authors contributed to the article and approved the submitted version.
