# Supplementary material for: Imaging mitochondria through bone in live mice using two-photon fluorescence microscopy with adaptive optics
Source: Front Neuroimaging. 2023 Feb 16;2:959601. doi: 10.3389/fnimg.2023.959601 (PMC10406258; doi:10.3389/fnimg.2023.959601)
Supplement: Supplementary file 1 [file Data_Sheet_1.docx]

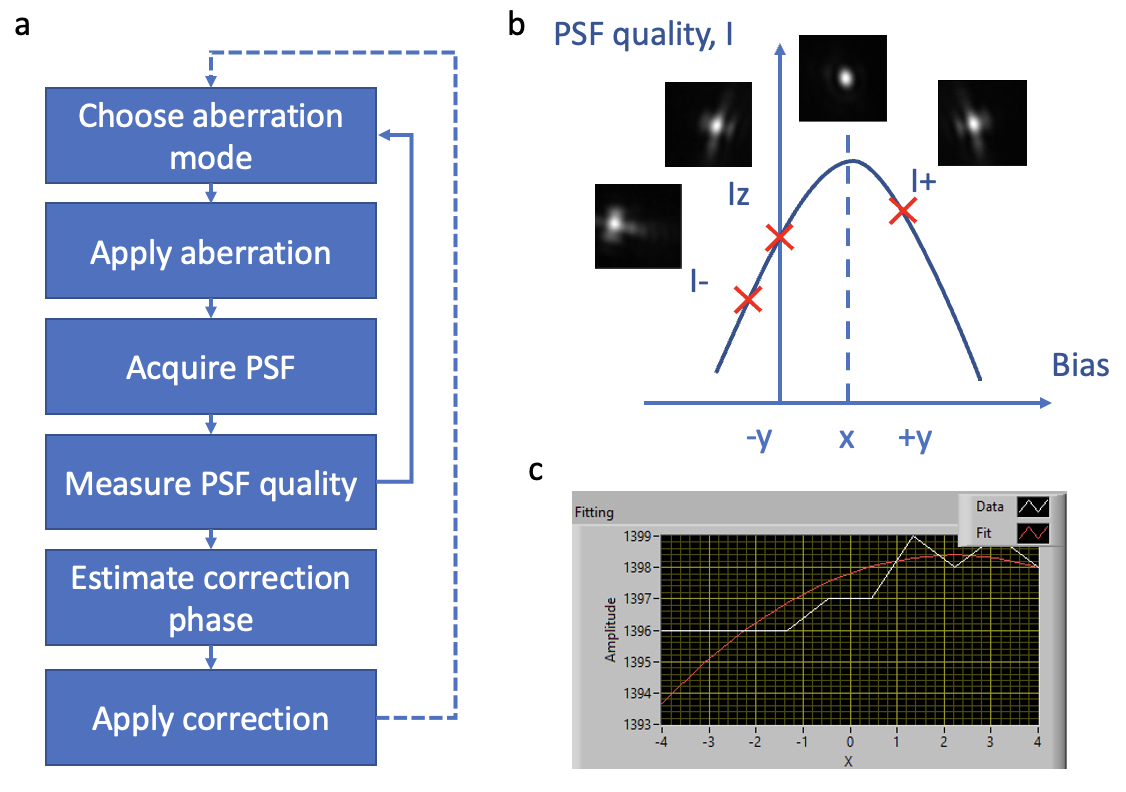


**Fig. S1.** PSF-intensity-metric-based wavefront sensing. (a) Flowchart depicting classical implementation of sensorless-AO-based wavefront measurement. (b) Sample PSF are captured for each mode using at least three different bias values (−$y$, $x$, +$y$). Image quality is estimated using a suitable defined metric for each image ($I$−,  $I$𝑧,  $I$+), and a quadratic function is fitted to the measured points. The peak value of the fitted curve corresponds to estimated best correction. Inset images represent a PSF affected by various amounts of Zernike mode. (c) Fitting function.


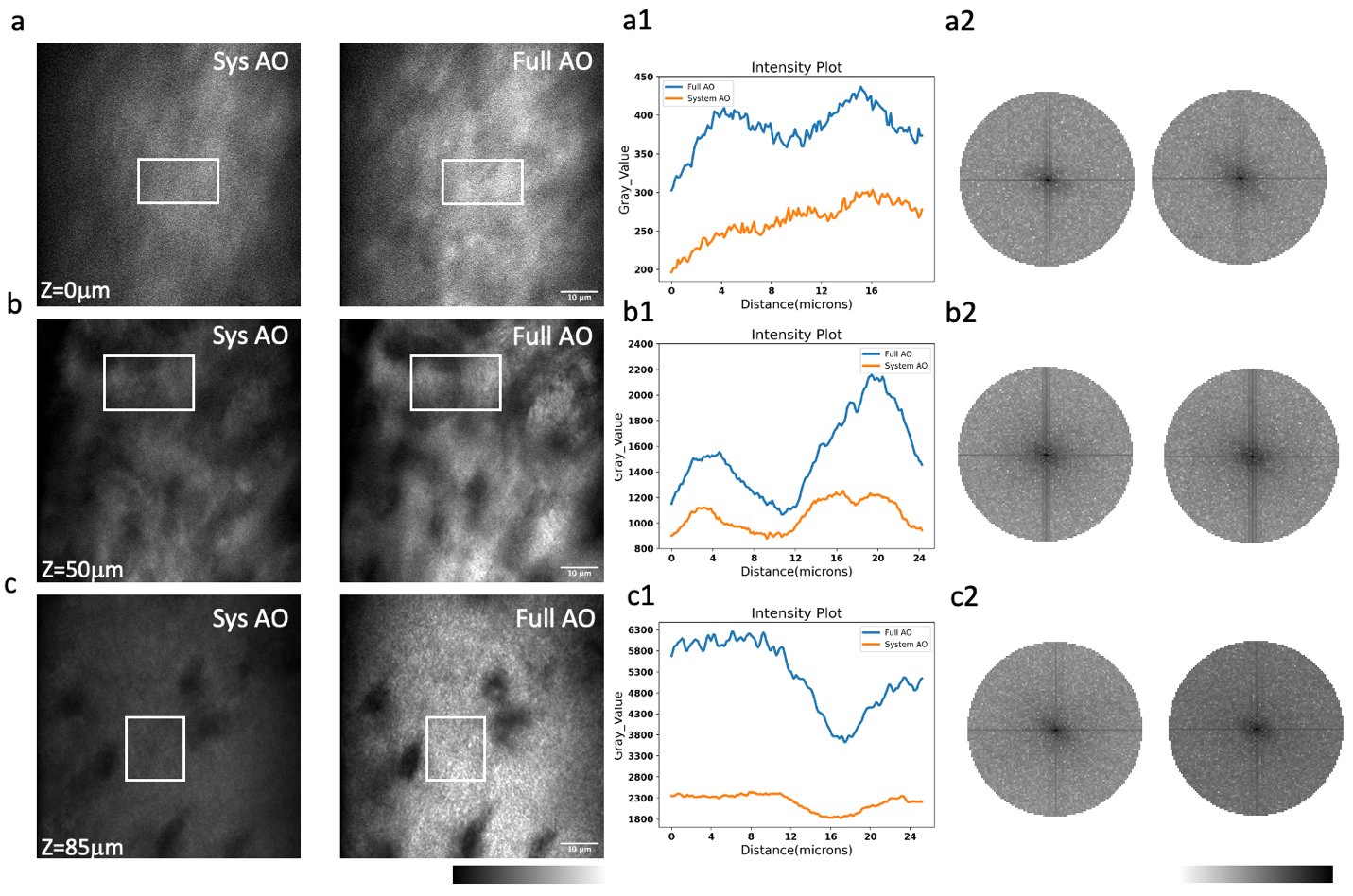


**Fig. S2.** Dynamic imaging of dendra-2 strained mouse brain marrow at the depth of 0, 50, 85μm with system AO (left) and with full AO (right) (a-c) in SHG channel. Corresponding signal profiles in the axial plane along the white blocks (a1-c1). FFT of SHG images with system AO (left) and with full AO (right) show in (a2-c2). The FOV is 67.5 $\mu m$ x 67.5 $\mu m$. Scale bar = 10 $\mu m.$


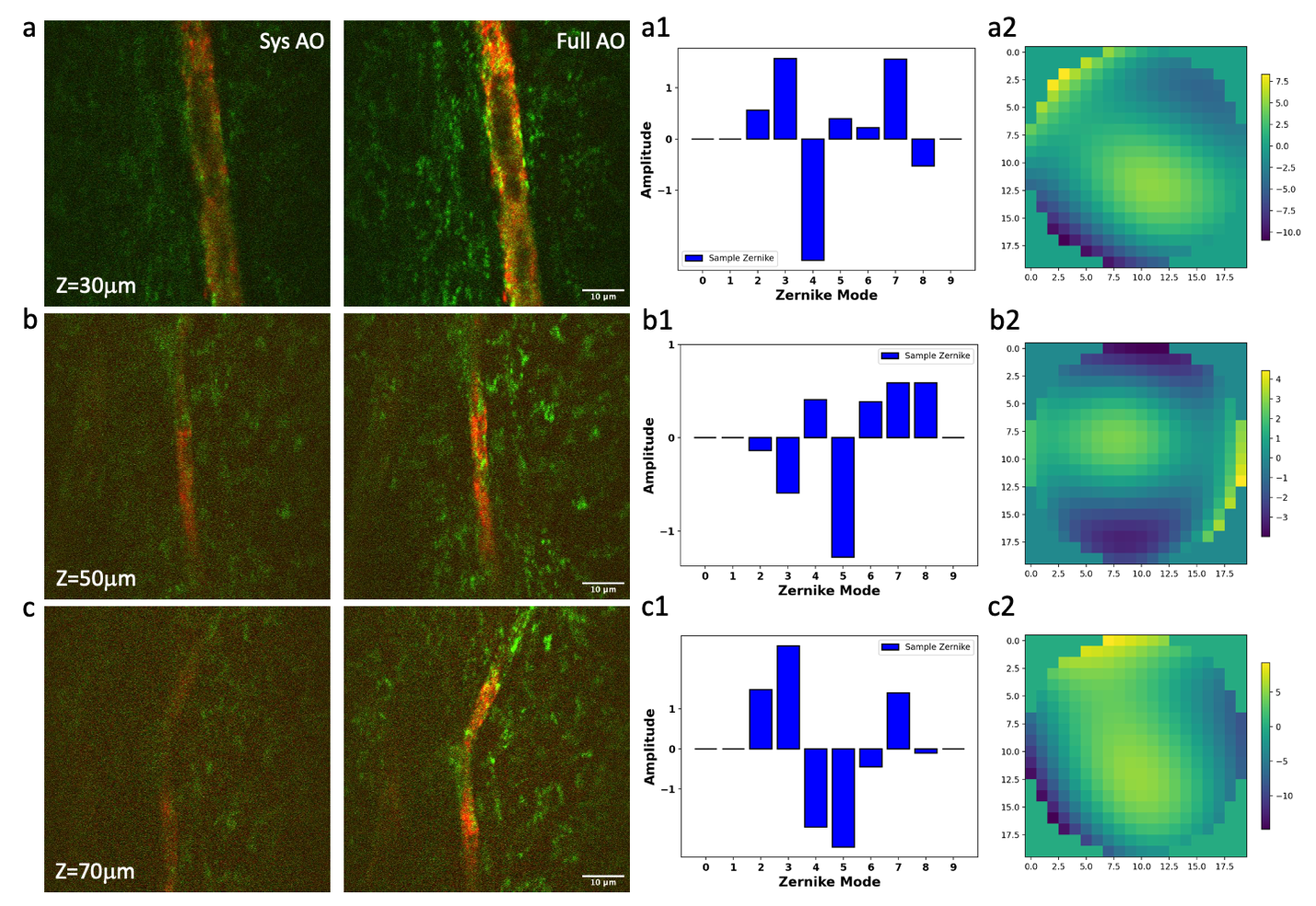


**Fig. S3.** Dynamic multichannel imaging of dendra-2 strained mouse blood vasculature and mitochondria at the depth of 30, 50, 70μm with system AO (left) and with full AO (right) (a-c). Wavefront after sample correction (a4-c4). Corresponding signal profiles of the red line (a1-c1) and FWHM profiles of the yellow line (a2-c2) and corresponding Zernike modes (a3-c3) for full AO. Images were acquired at the red square at 0μm, 50μm, 85μm depth(d). The FOV is 67.5 $\mu m$ x 67.5 $\mu m$. Scale bar = 10 $\mu m.$
